# Lymphoma presenting as autoimmune rheumatic disorder: clinical overlap and diagnostic considerations

**DOI:** 10.1097/MS9.0000000000004011

**Published:** 2025-10-07

**Authors:** Keysha González-Ramos, José M. Bretón, Luis M. Vilá

**Affiliations:** Division of Rheumatology, University of Puerto Rico Medical Sciences Campus, San Juan, Puerto Rico

**Keywords:** autoantibodies, hypocomplementemia, lymphoma, rheumatic disorders, rheumatic manifestations

## Abstract

Lymphomas are a heterogeneous group of malignancies arising from various lymphocyte subtypes, frequently presenting with systemic symptoms and multi-organ involvement. Their clinical features can closely resemble those of rheumatological diseases, presenting a significant diagnostic challenge. Rheumatic-like manifestations in patients with lymphoma may result from paraneoplastic syndromes – immune-mediated, indirect effects of the malignancy – or direct infiltration of joints and musculoskeletal tissues. Despite this overlap, there is a notable lack of structured, comprehensive guidance on how undiagnosed lymphomas may mimic rheumatological conditions. This review outlines the wide spectrum of rheumatological presentations associated with lymphoma, including inflammatory arthritis, hypertrophic osteoarthropathy, myositis, Paget’s disease, remitting seronegative symmetrical synovitis with pitting edema, and systemic features that resemble autoimmune disorders. These clinical manifestations are often accompanied by the presence of autoantibodies and hypocomplementemia, further complicating the diagnostic process. Distinguishing between primary rheumatological diseases and lymphoma-related manifestations is critical to avoid delays in diagnosis and the inappropriate use of immunosuppressive therapies, which may worsen an undetected malignancy. This review underscores the importance of a thorough diagnostic approach – including imaging, tissue biopsy, and advanced hematologic testing – before initiating or escalating treatment in patients with atypical, refractory, or rapidly progressive symptoms. By raising clinician awareness and encouraging continued research into improved diagnostic strategies, this work aims to facilitate earlier recognition of lymphomas masquerading as rheumatic diseases, ultimately improving patient outcomes.

## Introduction

Lymphomas represent a diverse group of lymphoproliferative disorders that differ in their lymphocyte origin, biological progression, clinical features, and outcomes^[1]^. These diseases arise from lymphocytes at various stages of maturation, and the specific characteristics of each lymphoma subtype are influenced by the type of cell from which they are derived – B cells, T cells, or natural killer (NK) cells^[1,2]^. There are more than 100 cell subtypes, per the latest World Health Organization classification from 2022^[2]^. From 2009 to 2013, the incidence of lymphoma in the United States was 22 per 100 000 people, accounting for about 5% of all cancers^[3]^. The median age at diagnosis is 63 years, with an overall 5-year survival rate estimated at 72%. They can impact any organ system and usually manifest as weight loss, night sweats, fevers, abnormal blood test results, skin lesions, neurological symptoms, and lymphadenopathy^[1,2]^.

**HIGHLIGHTS**

- Lymphoma can closely mimic rheumatic diseases, presenting with overlapping features such as musculoskeletal symptoms, systemic manifestations, and serological abnormalities that resemble those seen in autoimmune conditions.
- Clinicians should maintain a high index of suspicion for underlying lymphoma in patients who present with rheumatic-like symptoms, especially when there is an inadequate response to standard rheumatological therapies.
- A comprehensive differential diagnosis – including imaging studies, tissue biopsy, and hematological evaluations – is essential to avoid misdiagnosis and to prevent potential harm from initiating inappropriate treatments that could worsen an undetected malignancy.

Rheumatic symptoms can arise in the context of cancer, including lymphoma, either through direct mechanisms – such as tissue invasion or metastasis – or as distant paraneoplastic manifestations^[4,5]^. These symptoms may emerge at various stages of malignancy, sometimes serving as early indicators of an underlying cancer or appearing as late complications. This overlap is not unexpected, as shared immunopathogenic pathways – such as reduced Fas receptor expression or function, which may allow these autoreactive cells to evade elimination and contribute to disease development – support this link, with chronic inflammation playing a central role in the pathogenesis of both conditions^[6]^.

Delays in diagnosis and treatment can occur when a thorough evaluation for malignancy is not considered. Initiating immunosuppressive therapy for a presumed rheumatic condition may inadvertently accelerate the neoplastic process, and corticosteroid use has been associated with worse outcomes in patients with lymphoma. Consequently, rheumatologists must exercise caution when evaluating such cases. This review offers a comprehensive summary of the rheumatological clinical and serological manifestations observed in patients with lymphoma, as outlined in Supplemental Digital Content Table 1, available at: http://links.lww.com/MS9/A973 and Supplemental Digital Content Table 2, available at: http://links.lww.com/MS9/A974. It aims to enhance clinician awareness of the potential for lymphomas to present with features that mimic rheumatological diseases.

## Literature search strategy

A comprehensive literature search was conducted to identify relevant studies and case reports involving rheumatic and autoimmune manifestations associated with lymphoma. PubMed was utilized as the primary database due to its extensive coverage of biomedical literature. The search was restricted to English articles with full-text availability.

The search strategy incorporated a combination of Medical Subject Headings terms and free-text keywords, including: “lymphoma,” “paraneoplastic,” “autoimmune,” “arthritis,” “rheumatologic,” “serologies,” “rheumatic manifestations,” “rheumatic disorders,” “autoantibodies,” and “hypocomplementemia.” Boolean operators (and, or) were applied to refine the search and ensure the inclusion of studies spanning both lymphoproliferative disorders and rheumatological domains.

The initial search targeted literature published between 2000 and 2025, reflecting contemporary clinical understanding and diagnostic advancements. However, older publications were also reviewed selectively to capture atypical presentations that may not be reflected in more recent literature. Only articles involving patients diagnosed with lymphoma were included. Studies focusing on other hematological malignancies, such as multiple myeloma, myelodysplastic syndromes, acute and chronic leukemias, plasma cell dyscrasias, and myeloproliferative neoplasms, were systematically excluded to maintain specificity.

Reference lists of relevant articles were manually screened to identify additional reports meeting inclusion criteria. Preference was given to case reports, case series, and observational studies that provided detailed clinical, immunological, and serological data.

Artificial intelligence was employed solely to assist with the clarity, style, and wording of the manuscript in accordance with TITAN guidelines^[7]^. The work is entirely original, and all conceptual and substantive content was created by the authors.

## Musculoskeletal manifestations

### Inflammatory arthritis

Arthritis associated with hematological malignancies presents a wide spectrum of joint involvement patterns. Reported cases demonstrate variable presentations regarding the number of joints affected – ranging from monoarthritis (a single joint), oligoarthritis (2–4 joints), to polyarthritis (≥5 joints) – and the evolution over time, including migratory arthritis, where symptoms shift from one joint to another^[8–15]^. Elshahat *et al* evaluated rheumatological manifestations as the initial presentation of malignancies and found that such symptoms were significantly more common in hematological cancers (60%) than in solid tumors (20%). Among hematological malignancies, lymphomas were reported in 28% of cases, with arthralgia (joint pain) and arthritis (joint inflammation) being the most frequent rheumatologic presentations – appearing in 72% of malignancy-associated cases^[5]^.

In several reported cases presenting with monoarthritis, oligoarthritis, or polyarthritis, a final diagnosis of lymphoma was reached only after specific features during the diagnostic work-up raised suspicion and guided further investigation. Monoarthritis has been documented as a manifestation of lymphoma. This association is supported primarily by case reports. One case involved a man with elbow monoarthritis who was ultimately diagnosed with primary bone lymphoma, confirmed through bone histology^[10]^. Another case described a woman initially suspected of having prosthetic joint complications, who was later found to have primary synovial diffuse large B-cell lymphoma (DLBCL) based on surgical pathology^[16]^. A third case involved a patient with a 2-month history of persistent knee pain and swelling, whose diagnosis of non-Hodgkin’s lymphoma (NHL) was confirmed via arthroscopic synovial biopsy^[11]^. In addition to monoarthritis, Flower *et al* reported a case of adult T-cell leukemia/lymphoma presenting with oligoarthritis of the ankles, which later progressed to subcutaneous masses; the diagnosis was established through a biopsy of an abdominal mass. Among cases of lymphoma presenting with polyarthritis, Tsochatzis *et al* described a patient with symmetric, nondeforming, nonerosive inflammatory polyarthritis primarily affecting the small joints, wrists, knees, and ankles. This presentation preceded the diagnosis of angioimmunoblastic T-cell lymphoma (AITL) by 1 year. In their review of 16 cases, synovial fluid typically showed mild to moderate inflammation, while synovial biopsies ranged from nonspecific synovitis to malignant infiltration^[15]^. Similarly, Birlik *et al* reported a case initially diagnosed as rheumatoid arthritis (RA), which was ultimately revealed to be synovial B-cell lymphoma following finger amputation and biopsy for suspected osteomyelitis. Cosatti *et al* documented a patient presenting with polyarthritis, sicca symptoms, and rash mimicking RA, who was later diagnosed with malignant large B-cell lymphoma. Migratory polyarthritis has also been reported as a presenting feature of DLBCL^[14]^.

### Hypertrophic osteoarthropathy

Hypertrophic osteoarthropathy (HOA) is a paraneoplastic syndrome that can manifest as inflammatory arthritis and has been linked with various intrathoracic malignancies, including Hodgkin lymphoma (HL) and NHL^[17–19]^. Clinically, HOA is characterized by digital clubbing, periostitis and synovitis affecting joints distal to the long bones – typically the wrists, ankles, and knees. Gai *et al* reported a case of HOA in a young woman with intrathoracic HL and reviewed 13 additional cases since 1970, including pediatric patients, all associated with thoracic disease^[18]^. Similarly, Shapiro *et al* described three patients with intrathoracic HL who developed HOA, presenting with synovitis in distal joints – primarily the knees and ankles – and digital clubbing in 67% of cases^[20]^. Supporting these findings, a retrospective review by Meyer *et al* identified six cases of secondary HOA related to malignancy, including both NHL and HL, with all lymphoma cases involving the thoracic cavity^[21]^. Given these observations, the presence of distal synovitis accompanied by digital clubbing or periostitis should prompt a thorough evaluation of the chest for possible intrathoracic lymphoma. Early imaging, along with biopsy when indicated, is essential for achieving an accurate and timely diagnosis.

### Myalgia and myositis

The association between idiopathic inflammatory myopathies and malignancy, including lymphoma, is well established^[22–27]^. However, lymphoma itself may also present with myalgia and myositis, closely mimicking the clinical features of inflammatory myopathy. A notable case involved a 21-year-old woman with persistent myalgia and proximal muscle weakness, initially suggestive of idiopathic myositis, who was ultimately diagnosed with stage IV nodular sclerosis classical HL involving muscle and bone, confirmed by imaging and lymph node biopsy^[25]^. Similarly, in a 10-year study involving 15 142 patients admitted to a general hospital, Naschitz *et al* identified one patient whose presentation with localized myositis preceded a diagnosis of HL by 12 months^[28]^. Other case reports describe subacute panniculitis-like T-cell lymphoma presenting with clinical and pathological features of dermatomyositis^[29]^, while NK/T-cell lymphomas have been reported to cause phlegmonous myositis of the lower extremity^[30]^ as well as inflammatory myositis^[31,32]^. DLBCL has also been shown to cause focal myositis, presenting as a forearm mass^[33]^. These cases underscore the importance of considering occult lymphoma in the differential diagnosis of patients presenting with inflammatory myopathy, particularly when clinical features are atypical.

### Paget’s disease

Paget’s disease, also known as osteitis deformans, is a chronic skeletal disorder characterized by abnormal and excessive bone remodeling. It affects approximately 3–4% of individuals over the age of 40 and is the second most common bone disease in the aging U.S. population, following osteoporosis^[34]^. Malignant transformation in Paget’s disease is rare. Osteosarcoma is the most frequent malignancy, accounting for 50–60% of cases, followed by malignant fibrous histiocytoma or fibrosarcoma (20–25%), chondrosarcoma (10%), and, less commonly, lymphoma and angiosarcoma (1–3%)^[34]^. Imaging findings of lymphoma involving bone can closely resemble those seen in Paget’s disease, which typically presents with diffuse sclerosis and bone deformities that may mimic skeletal metastases or lymphoma on radiographs. This radiological overlap can result in misdiagnosis, emphasizing the importance of thorough clinical correlation and maintaining a broad differential diagnosis. Notably, a case of B-cell lymphoma was misdiagnosed as Paget’s disease for over 2 years, delaying appropriate treatment and illustrating the potential consequences of relying on imaging alone without comprehensive evaluation^[35]^.

### Remitting seronegative symmetrical synovitis with pitting edema

Remitting seronegative symmetrical synovitis with pitting edema (RS3PE) is a rare and often underrecognized condition, largely due to the absence of definitive diagnostic criteria and limited clinical awareness^[36]^. RS3PE has been associated with underlying malignancies, including NHL, although such cases remain relatively uncommon^[17,36,37]^.

## Systemic manifestations mimicking rheumatic disorders

Lymphoma can present with systemic manifestations that are also frequently observed in autoimmune rheumatic diseases such as necrotizing granulomatosis, pulmonary and central nervous system (CNS) involvement, and IgG4-related disease (IgG4-RD). Rheumatological diseases that manifest with necrotizing granulomatosis encompasses sarcoidosis, granulomatosis with polyangiitis (GPA), eosinophilic granulomatosis with polyangiitis (EGPA), and rheumatoid nodules. Granulomatous lung diseases are diagnostically challenging due to their nonspecific nature and diverse causes, with definitive diagnoses often requiring the identification of causative agents, which is not always possible.

### Necrotizing granulomatosis – sarcoidosis

Sarcoidosis and infections such as tuberculosis account for a significant proportion of granulomatous diseases. Granulomas, which are organized collections of mononuclear phagocytes, may contain necrosis or inflammatory cells; however, the presence or absence of necrosis is not always indicative of an infectious etiology^[38]^. Non-necrotizing granulomas are typically associated with noninfectious conditions, such as sarcoidosis. A notable link exists between pulmonary granulomas and malignancy, particularly in cases where granulomas form adjacent to lung carcinomas or within regional lymph nodes, often as an immune response to tumor antigens. Granulomatous reactions have also been observed in various cancers, including lymphomas^[38]^. In fact, granulomatous inflammation has been reported in approximately 13.8% of HL cases and 7.3% of NHL cases^[39]^.

Sarcoidosis or sarcoid-like reactions may occur before, during, or after a cancer diagnosis, which can complicate the distinction between cancer-associated granulomas and true sarcoidosis^[38,40]^. Wu *et al* described seven cases in which the diagnosis of lymphoma was delayed due to varied histologic presentations. In all instances, granulomatous inflammation coexisted with neoplastic cells. Clinical presentations included constitutional symptoms such as fever, weight loss, abdominal pain, and dyspnea^[41]^. Histopathological evaluation of biopsies from the liver, spleen, bone marrow, mediastinal lymph nodes, or skin revealed non-necrotizing epithelioid granulomas in most patients, while one case demonstrated partially necrotic granulomas with multinucleated giant cells. Initially, these patients were misdiagnosed with conditions such as noncaseating granulomatous inflammation, mycobacterial infections, reactive T-cell hyperplasia, granulomatous mycosis fungoides, or other reactive granulomatous disorders^[41]^. Subsequent follow-up, however, confirmed a diagnosis of lymphoma. These findings highlight the potential for both necrotizing and non-necrotizing granulomas to mimic infections, sarcoidosis, or autoimmune diseases such as vasculitis. As a result, lymphoma presenting in this manner may be misdiagnosed, leading to delayed recognition and treatment.

### Necrotizing granulomatosis – GPA

GPA is characterized by necrotizing granulomatous inflammation typically affecting the upper respiratory tract, lungs, and kidneys. Its clinical and radiological features can closely overlap with those of lymphoma, creating significant diagnostic challenges^[42]^. For example, a case report describes a young male with high-grade Epstein–Barr virus-associated DLBCL who presented with necrotizing granulomas, pulmonary nodules, and elevated inflammatory markers – findings that initially mimicked GPA^[43]^. Similarly, NK/T-cell lymphomas frequently involve the upper aerodigestive tract, including the nasal cavity and paranasal sinuses, and may be mistaken for GPA due to their similar anatomical involvement and clinical presentation^[30]^.

### Necrotizing granulomatosis – EGPA

Constitutional symptoms accompanied by pulmonary or cutaneous involvement in B-cell or T-cell lymphomas can closely mimic EGPA, particularly when histological findings reveal granulomas and/or necrotizing vasculitis, along with overlapping radiologic features^[44]^. One reported case involved a 69-year-old man with a history of chronic sinusitis, nasal polyps, and asthma who was initially diagnosed with EGPA after presenting with weakness, paresthesia, and fever, along with laboratory findings of eosinophilia and a positive anti-neutrophil cytoplasmic antibodies (ANCA) test. However, further diagnostic evaluation, including tissue biopsy, revealed the true diagnosis of low-grade B-cell lymphoma. The patient responded well to chemotherapy and achieved a favorable clinical outcome^[45]^.

### Pulmonary involvement

Lung involvement occurs in 15–40% of HL cases, but primary pulmonary Hodgkin’s lymphoma (PPHL) is extremely rare, comprising less than 0.5% of primary lung cancers^[46]^. A 53-year-old woman with progressive respiratory symptoms and radiological lung changes was initially suspected of having GPA, but after further investigation, including a lung biopsy, she was diagnosed with PPHL, confirmed through immunohistochemistry showing atypical Hodgkin’s-like cells^[46]^. Also, PPHL has been mistaken for RA-associated organizing pneumonia (OP). A 61-year-old man with a 30-year smoking history presented with chronic cough, fever, and joint pain for 2 years, showing migratory lung consolidations that improved with antibiotics or corticosteroids. After negative tests for infections, autoimmune markers, and tumors, a lung biopsy initially suggested OP, and he was diagnosed with seronegative RA-associated OP. However, follow-up computed tomography (CT) and positron emission tomography (PET) scans revealed persistent lung lesions, leading to a second biopsy that identified atypical lymphoid cells and immunohistochemical staining confirming PPHL^[47]^.

### CNS involvement

Lymphoma can involve the CNS, often mimicking a variety of rheumatological and autoimmune conditions, which presents significant diagnostic challenges. In one case, a patient was initially diagnosed with neuropsychiatric systemic lupus erythematosus (SLE) based on stroke-like symptoms and abnormal brain magnetic resonance imaging (MRI) findings. Despite treatment with corticosteroids and intravenous immunoglobulin, the symptoms persisted but later improved with rituximab, ultimately leading to a diagnosis of primary CNS lymphoma confirmed by brain biopsy^[48]^. Another case involved a young immunocompetent woman with progressive cognitive deficits and multiple brain lesions on MRI. An extensive work-up for autoimmune, genetic, and infectious causes was unremarkable, but a brain biopsy revealed primary CNS T-cell lymphoma, confirmed through T-cell receptor gene rearrangement, despite initial suspicion of vasculitis^[49]^. Similar cases of CNS lymphoma mimicking primary CNS angiitis have also been reported^[50,51]^. In another instance, a 49-year-old man was misdiagnosed with neurosarcoidosis based on clinical features including cough, bilateral hilar lymphadenopathy, S1 radiculopathy, and facial nerve palsy. His condition deteriorated despite immunosuppressive therapy, and a biopsy of newly developed cutaneous nodules ultimately revealed DLBCL involving both cranial and peripheral nerves^[52]^. A separate case described a patient with a history of acute uveitis who was initially diagnosed with neuro-Behçet’s disease based on characteristic brain lesions. However, following treatment with high-dose methotrexate, the lesion persisted, and a biopsy confirmed the diagnosis of CNS peripheral T-cell lymphoma presenting as lymphomatosis cerebri^[53]^.

These cases underscore the importance of considering lymphoma in the differential diagnosis when CNS symptoms mimic systemic inflammatory disorders. Although imaging is useful, it is often nondiagnostic. CNS lymphomas typically present as single or multiple parenchymal lesions located in the supratentorial region – especially the basal ganglia, periventricular areas, midline structures, and corpus callosum – and may also involve the brain hemispheres, leptomeninges, or subependymal regions. The involvement of the posterior fossa or spinal cord is rare^[54]^. Ultimately, biopsy remains essential for accurate diagnosis and appropriate management.

### IgG4-related disease

Although IgG4-RD is frequently described in the literature as a mimicker of lymphoma, there are also reported cases in which lymphoma was initially misdiagnosed as IgG4-RD^[55,56]^. One such case involved an elderly man who presented with elevated serum IgG4 levels, lymphadenopathy, and a retroperitoneal mass. He was initially treated for presumed IgG4-RD, but a subsequent biopsy of the mass revealed an underlying lymphomatous process^[57]^. In two other cases, patients were initially diagnosed with Mikulicz’s disease based on elevated IgG4 levels and bilateral enlargement of the parotid and submandibular glands. However, histopathological examination ultimately confirmed the presence of marginal zone B-cell lymphoma in both instances^[58,59]^. These cases highlight the potential for diagnostic uncertainty and underscore the need for tissue biopsy and careful histological evaluation when clinical features overlap between IgG4-RD and lymphoproliferative disorders.

## Serological features mimicking rheumatic disease

Serological features commonly associated with autoimmune rheumatic diseases can also occur in patients with lymphoma, contributing to diagnostic confusion. The most frequently reported abnormal serologies include RF^[15,60,61]^ and ANA^[62–64]^, with ANA positivity observed in up to 21% of lymphoma cases^[64]^. Elevated anti-double-stranded DNA antibodies and hypocomplementemia have also been reported, often leading to a misdiagnosis of SLE^[63]^. In cases of AITL, the presence of multiple autoantibodies – including anti-Ro/SSA, anti-La/SSB, anti-Jo-1, anti-ribonucleoprotein, and anti-histone antibodies – can further complicate diagnosis^[61]^. Li *et al* described a case of AITL presenting with polyarthritis and cryoglobulinemic nephritis, along with hypocomplementemia, elevated ANA, and antiphospholipid antibodies; the patient was initially misdiagnosed and unsuccessfully treated with methylprednisolone, plasma exchange, and rituximab. A separate report reviewing 16 cases found autoantibodies in 25% of patients with AITL-associated arthritis, including ANA and anti-smooth muscle antibodies^[15,65]^.

Cohort studies have demonstrated an association between abnormal autoantibody formation and lymphoma. In a retrospective cohort study by Barreno *et al*, various autoantibodies – including lupus anticoagulant, ANA, and ANCA – were detected among 30 patients with NHL. One patient had elevated antibodies against proteinase 3 (PR3) and myeloperoxidase (MPO), ultimately leading to a diagnosis of stage 3B high-grade NHL after splenectomy^[66]^. Notably, lymphoma has been associated with ANCA positivity, including both PR3 and MPO, even in the absence of clinical vasculitis^[43]^. Additional reports have described NK/T-cell lymphoma mimicking GPA, with paranasal and renal involvement and positive p-ANCA and PR3 antibodies; a definitive diagnosis was made only after genetic and molecular analysis of a kidney biopsy revealed malignancy^[67]^. The presence of anti-TIF-gamma antibodies has also been observed in association with marginal zone lymphoma, suggesting its potential as a biomarker and its involvement in the autoimmune responses associated with lymphomagenesis^[68]^.

Hypocomplementemia has been identified in certain lymphomas, including immunoblastic lymphadenopathy and immunoblastic lymphadenopathy-like T-cell lymphoma, often coexisting with hematological abnormalities such as anemia and thrombocytopenia^[69]^. In DLBCL, hypocomplementemia and hypogammaglobulinemia have been associated with advanced disease stages and poorer clinical outcomes, likely reflecting broader immune dysfunction that impairs the host’s capacity to control tumor progression^[70]^. In autoimmune conditions such as primary Sjögren’s syndrome, hypocomplementemia is considered a marker of increased morbidity and a heightened risk of lymphoma, supporting the hypothesis that chronic immune activation and complement consumption may contribute to the pathogenesis of lymphoma^[71]^.

Neoplastic diseases utilize the complement system to evade immune detection, promote tumor growth, and enhance their surrounding environment for proliferation^[72]^. Recent research in neuroscience highlights the complement system’s role in detecting stress signals, regulating immune responses, and influencing neuroplasticity. In primary CNS lymphoma, studies show that complement proteins like C1q and Factor H are upregulated in the cerebrospinal fluid, which may suppress immune activity and create a tumor-friendly microenvironment^[72]^. These results highlight the broad variability in autoantibody profiles among patients with lymphoma. This reinforces the importance of correlating these findings with clinical symptoms, prior medical history, and a thorough physical examination to ensure accurate diagnosis and management.

## Diagnostic approach

**Figure 1.**
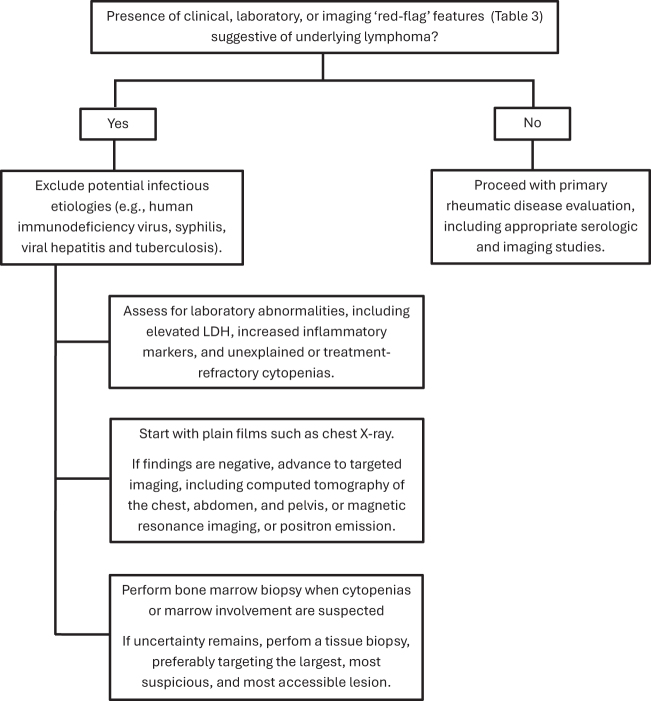
Diagnostic approach to suspected underlying lymphoma in patients with rheumatic manifestations.

The diagnosis of underlying lymphoma in patients presenting with atypical rheumatic manifestations can be particularly challenging. Figure 1 illustrates a diagnostic approach for evaluating these patients. While many paraneoplastic features may mimic primary rheumatic diseases, the presence of specific clinical, laboratory, and imaging “red flags” should raise suspicion and prompt further evaluation^[73]^. Worrisome clinical signs include severe constitutional or “B” symptoms, as well as lymphadenopathy, particularly when nodes are firm, greater than 2 cm in size. Additional red flags include rapidly progressive symptom onset, atypical joint involvement, or resistance to standard immunosuppressive or anti-rheumatic therapy, among others. Laboratory abnormalities that may support suspicion of lymphoma include elevated lactate dehydrogenase, hypocomplementemia without a clear autoimmune correlate, multiple autoantibodies inconsistent with the clinical presentation, and unexplained or treatment-refractory cytopenias. Disproportionately elevated inflammatory markers may also warrant further investigation. Imaging findings such as pulmonary nodules, mediastinal lymphadenopathy, periostitis, or abnormal PET scan uptake may indicate a lymphoproliferative process. The complete list of these “red flags” is presented in Supplemental Digital Content Table 3, available at: http://links.lww.com/MS9/A975.

In patients with suspected underlying lymphoma, further investigations should include laboratory testing for common infectious diseases such as human immunodeficiency virus, syphilis, viral hepatitis, and tuberculosis, as these conditions may mimic lymphoproliferative disorders or should be assessed prior to initiating lymphoma treatment.

In select cases, bone marrow evaluation or advanced imaging may be required to complete the diagnostic work-up. If uncertainty persists, a tissue biopsy should be performed, ideally targeting the largest, most suspicious, and most accessible area.

## Diagnostic utility of tissue biopsy

The choice of biopsy site is critical and should be guided by the clinical presentation^[74]^. Lymph nodes, bone marrow, or involved extranodal sites, such as the synovium or salivary glands, may be appropriate targets. Importantly, adequate tissue sampling is required, as limited or crushed samples may hinder accurate classification. A comprehensive diagnostic work-up should include routine histopathology, immunohistochemistry, flow cytometry, and, when indicated, molecular studies. These components are crucial for establishing the correct diagnosis and guiding treatment decisions, highlighting the importance of early collaboration with hematopathology when lymphoma is suspected.

While current diagnosis relies heavily on histopathology, emerging technologies such as liquid biopsies (e.g., circulating tumor DNA [ctDNA]), gene expression profiling, and advanced imaging biomarkers hold promise in improving early detection and in differentiating lymphoma from autoimmune mimics^[75]^. Liquid biopsy approaches that analyze ctDNA have quickly evolved from experimental techniques to valuable clinical biomarkers in lymphoid malignancies, including lymphoma with rheumatological presentations. Their minimally invasive nature allows for repeated sampling, providing dynamic insights into tumor burden, genetic diversity, and treatment response that may be overlooked by conventional imaging or tissue biopsy alone. When combined with established imaging tools such as positron emission tomography/computed tomography or MRI, ctDNA analysis can improve risk assessment and help tailor therapy in a more personalized and timely manner. Nonetheless, challenges remain, and there is a need for standardized, reproducible assays and validation across diverse clinical settings.

## Conclusion

Lymphoma can present with clinical features that closely mimic new-onset rheumatic diseases, posing a significant diagnostic challenge for rheumatologists. The overlap in manifestations – such as arthritis, myositis, systemic inflammation, and autoantibodies – may lead to misdiagnosis, particularly when these manifestations resemble those of autoimmune or inflammatory conditions. It is therefore essential for rheumatologists to maintain a high index of suspicion for an underlying malignancy, especially in patients who fail to respond to standard rheumatological therapies as expected.

A meticulous approach to differential diagnosis is imperative. Lymphoma should be considered in patients presenting with atypical features or resistance to conventional treatment. A comprehensive diagnostic workup – including imaging studies, tissue biopsy with specialized staining techniques, and advanced hematological assessments – should be performed prior to initiating or escalating immunosuppressive therapy. These hematological evaluations may include flow cytometry for immunophenotyping, molecular and cytogenetic testing for genetic abnormalities, and thorough bone marrow analysis. This precautionary strategy is crucial to avoid initiating immunosuppressive treatments that could inadvertently mask or exacerbate an undiagnosed lymphoma, thereby compromising patient outcomes and increasing the risk of further complications.

## Data Availability

Data availability statement does not apply, as this is a review article.

## Research registration unique identifying number (UIN)

UIN was not required, as this is a review article.
